# Fast and Sensitive Voltammetric Method for the Determination of Rifampicin on Renewable Amalgam Film Electrode

**DOI:** 10.3390/s21175792

**Published:** 2021-08-28

**Authors:** Marek Szlósarczyk, Robert Piech, Anna Milc, Urszula Hubicka

**Affiliations:** 1 Department of Inorganic and Analytical Chemistry, Medical College, Jagiellonian University, Medyczna 9, 30-688 Kraków, Poland; a.milc@wp.pl (A.M.); urszula.hubicka@uj.edu.pl (U.H.); 2 Faculty of Materials Science and Ceramics, AGH University of Science and Technology, Al. Mickiewicza 30, 30-059 Kraków, Poland; rpiech@agh.edu.pl

**Keywords:** rifampicin, pharmaceutical analysis, mercury film electrodes, voltammetry

## Abstract

In this work, a new sensitive voltammetric method for the determination of rifampicin without time-consuming preconcentration is presented. The objective was to develop a simple, fast and sensitive voltammetric procedure for the analysis of rifampicin in pharmaceutical products. The cyclic renewable mercury film silver-based electrode (Hg(Ag)FE) was applied as a working electrode for this purpose. The optimal conditions for the determination of rifampicin were defined, in terms of the composition of supporting electrolyte (including pH) and instrumental parameters (potential and time of deposition, step potential, pulse height). The method was validated resulting in a satisfactory linearity range of 0.4–250.0 µgmL^−1^; the limits of detection and quantification are 0.12 µgmL^−1^ and 0.4 µgmL^−1^, respectively; and the repeatability of the method expressed as RSD is 4.1% (n = 6) with a surface area of 10.9 mm^2^. The proposed method was successfully applied in the analysis of rifampicin in simple and composed pharmaceutical formulations.

## 1. Introduction

Rifampicin (RIF) is a macrocyclic antibiotic, recommended as the first-line treatment of tuberculosis, also with other therapeutic agents [1], in the prevention of the *Neisseria meningitidis* infection [2] and even for antibiotic prophylaxis of the postoperative *endophthalmitis* [3]. Considering reported variability of RIF pharmacokinetics, therapeutic ineffectiveness was explained by its low plasma concentrations [4,5]. Problematic or delayed achievement of RIF therapeutic concentration may also contribute to the death of patients suffering from pulmonary tuberculosis. Moreover, several studies have shown a close relationship between RIF toxicity and its high plasma level [6]. Considering this variability of RIF, the therapeutic drug monitoring of RIF is helpful in tuberculosis therapy to prevent low plasma concentrations, drug-related toxicity, therapeutic failure and drug resistance or tuberculosis relapse [4,5].

Tuberculosis (TB) is one of the major life-threatening infectious diseases, millions of people continue to fall sick with TB each year. According to the WHO data, 10 million new cases of TB (including 1.2 million children) were reported, and 1.4 million people finally died from TB in 2019 [7]. Due to a considerable increasing rate of rifampicin resistance (RR) among strains of *Mycobacterium tuberculosis* (206k cases of multi-drug resistance MDR/RR-TB were detected and reported in 2019) combined with the evidence on the interaction between COVID-19 and TB in particular as public health implications (lower access to health services) [8,9]. A recent WHO report on tuberculosis suggested urgent actions to improve coverage and quality of treatment for people with drug-resistant TB and better insight into MDR tuberculosis development. [7]. One of the factors which can help in this case is active ingredient analysis, necessary in drug quality control and as well as a useful tool in therapeutic drug monitoring during the treatment of tuberculosis [5]. 

According to the available literature, the following analytical methods are commonly used for the determination of rifampicin in pharmaceuticals and body fluids: separation techniques—thin layer chromatography [10] with densitometric detection [11] or high-performance [12], high-performance and ultra-high-performance liquid chromatography [13], micellar electrokinetic chromatography [14], capillary electrophoresis [15] and combined high-performance liquid chromatography-tandem mass spectrometry [16]; optical techniques—spectrophotometry [17] and electroanalytical techniques. In the case of electroanalysis, stripping techniques are used preferably such as potentiometry and voltammetry [18,19,20] but in most cases voltammetry are limited to a positive potential range. The analysis in the negative potential range is possible using classical mercury electrode with high sensitivity, reproducibility and linearity range. However, the toxicity of mercury limits the application of the mercury electrodes in the analysis in both laboratory and portable applications [21]. One of the approaches to overcoming these limitations is to use a renewable silver amalgam film electrode (Hg(Ag)FE). A detailed description of the mentioned sensor and the first application was described previously [22]. The simple design of the Hg(Ag)FE electrode combined with the possibility of refreshing electrode surface provides a broad range of applications. Moreover, the use of the Hg(Ag)FE allows sensitive voltammetric determination of both inorganic and organic compounds, including active pharmaceutical ingredient (API) in various matrices [23,24,25,26,27,28,29]. 

In this work, the application of cyclic renewable mercury film silver-based electrode (Hg(Ag)FE) in the determination of rifampicin is presented. The method based on differential pulse voltammetric technique was performed without preconcentration time allowing detection of rifampicin at trace level. The new method was examined with a successful application for the determination of rifampicin contents as API in several simple and composed pharmaceutical formulation. 

## 2. Materials and Methods

### 2.1. Measuring Apparatus and Software

All the voltammetric measurements were carried out at room temperature, using a multipurpose Electrochemical Analyzer M161 (MTM-ANKO, Kraków, Poland) controlled by PC. The typical three-electrode system of the electrode stand M164 (MTM-ANKO, Kraków, Poland) used Hg(Ag)FE as a working electrode, an Ag/AgCl reference electrode with a double junction and a platinum wire as an auxiliary electrode. SevenCompact pH meter S220 (Mettler-Toledo, Geneva, Switzerland) was used for pH measurements. Stirring was performed using a magnetic bar rotating with a speed of 500 rpm. For all the dataset processing and statistical calculations Statistica 13.3 (Tibco Software Inc., Palo Alto, CA, USA), OriginPro 2020 (OriginLab Corporation, Northampton, MA, USA) and MarvinSketch (ChemAxon, Budapest, Hungary) were used.

### 2.2. Chemicals and Glassware

All reagents of analytical grade: CH_3_COOH (Suprapur), mercury GR for polarography, Triton X-100 and were from Merck (Darmstadt, Germany). A standard rifampicin solution of (10.3 mg/5 mL) and lower concentrations were prepared by dissolving rifampicin (Sigma-Aldrich, Steinheim, Germany) or by appropriate dilution in quadruple distilled water and were stored in a refrigerator. The silver wire (Ø 0.5 mm) for the film electrode was made of 99.99% polycrystalline silver (Goodfellow Science Park, Huntingdon, England). Before use, all glassware was immersed in a diluted (1:10) aqueous solution of HNO_3_ (65%) and then rinsed copiously with distilled water.

### 2.3. Standard Procedure of Measurements

Differential pulse voltammetry (DPV) was chosen for quantitative measurements of RIF using and the standard addition procedure. Before each measurement mercury film of Hg(Ag)FE electrode was refreshed and after conditioned applying a potential of −400 mV for 3 s. The potential of Hg(Ag)FE was changed in the following order: conditioning/starting potential −400 mV and recording in the cathodic direction from −400 mV to −1050 mV. The DPV instrumental parameters were as follows: step potential, 4 mV; pulse potential, 30 mV; time step potential, 20 ms (10 ms of waiting and sampling time) in 0.1 M CH_3_COOH as supporting electrolyte.

### 2.4. Analysis of Rifampicin in Pharmaceutical Formulation

Ten tablets of Rifampicyna TZF^®^ and ten Rifamiazid^®^ (Polfa Tarchomin both) capsules content was weighed and powdered separately in an agate mortar, and the average weight of the tablet and capsule were calculated. Next, an appropriate amount of the powdered material was weighed and transferred to a volumetric flask and filled with quadruple distilled water to achieve the desired concentration. The obtained solution after 20 min of sonication, was filtered through a 0.45 µm membrane disk and filled up to the mark in the 10 mL volumetric flask. After, the obtained solution was ready to direct measurements, according to the proposed procedure, without additional pretreatment or extraction steps. 

## 3. Results

### 3.1. Influence of Supporting Electrolyte Type and pH on Rifampicin Peak

**Figure 1:**
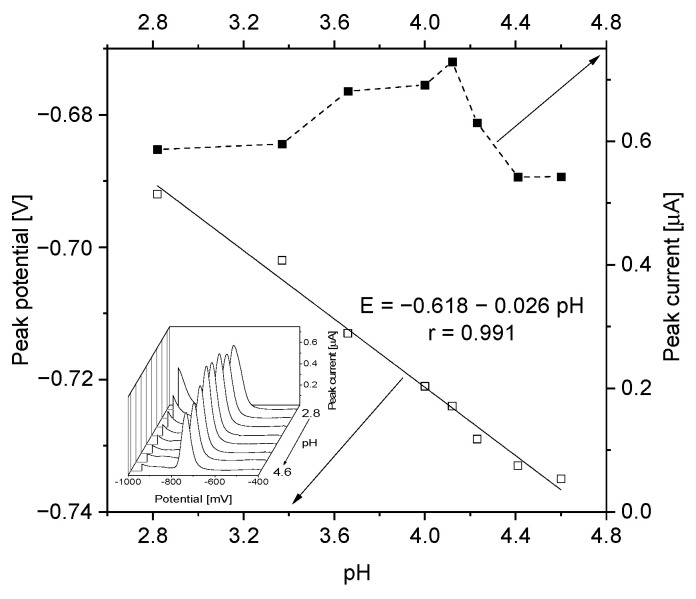
Dependence of peak current and potential on pH value. Instrumental parameters: ΔE = 30 mV, Es = 4 mV, tw, tp = 10 ms. Stirring rate, 500 rpm.

The effect of various supporting electrolytes on RIF peak was studied including the solutions of salts (potassium chloride and nitrate), base (sodium hydroxide), acid (acetic and phosphoric) and buffers (Britton—Robinson, phosphate, acetate). The height and shape of the peaks (based on the FWHM value—full width at half maximum) as well as the stability and repeatability of the signal were adopted as the evaluation criteria. In the investigated range, the pH influenced RIF peak current and potential causing its widening and enlarging. Although, the peak potential was shifted from −692 mV to −735 mV and the relationship with pH has a linear characteristic. In the case of RIF, the optimal pH ranged from 3.3 to 4.1, while outside these values its peak current was decreasing (Figure 1). The best RIF peak shape, good signal-to-noise ratio (SNR) and optimal ionic medium conductivity were achieved for the solution of 0.1 M CH_3_COOH (pH = 3.3) and this concentration was chosen for further analysis. Considering the protonation of RIF, the optimal pH was close to the theoretical value calculated for these conditions at a given pH.

### 3.2. Effect of the DPV Parameters on Rifampicin Peak

**Figure 2:**
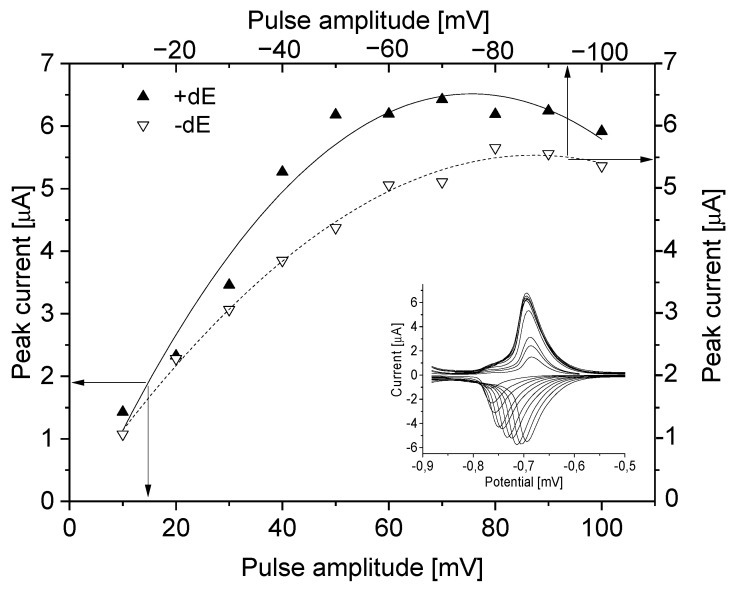
Pulse amplitude (from 10 mV to 100 mV) dependence on peak current in negative (---) and positive (—) mode. The electrode surface area 9.7 mm^2^. Instrumental parameters: Es = 4 mV, tp, tw = 10 ms, stirring rate 500 rpm.

Using the DPV technique RIF showed two peaks at potentials −693 mV and −966 mV in CH_3_COOH (pH = 3.3). The keys parameters of the DPV technique are pulse amplitude (dE), potential step amplitude (Es), waiting (tw) and sampling time (tp). So, the effect of the above instrumental parameters has been investigated for the first peak (twice as high as the second) in the ranges: 10–100 mV (both +/−) for dE, 1–9 mV for Es and 5–40 ms for both tw and tp. The increase of the pulse amplitude in negative mode caused peak shifting from −763 mV to −694 mV (Figure 2). The pulse amplitude above 40 mV caused a significant reduction of SNR due to the growth of background current and decrease of peak current (above 70 mV). At the optimal pulse amplitude of 30 mV, the RIF peak current was equal to 3.48 µA and showed the best SNR value.

**Figure 3:**
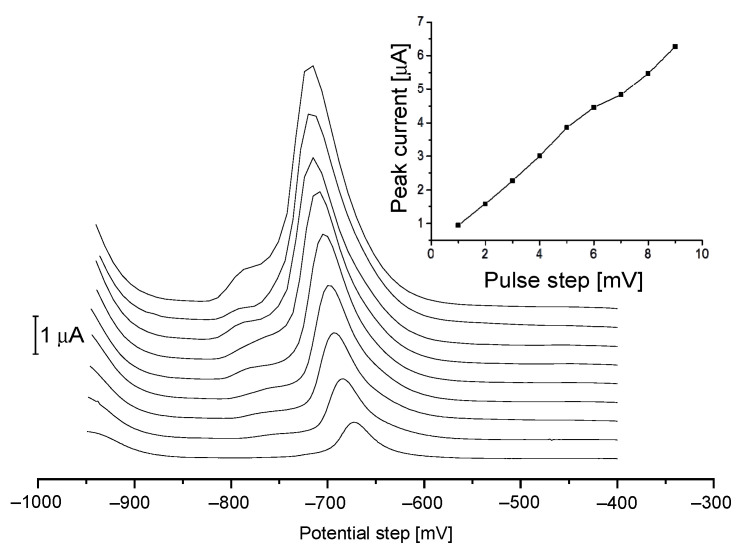
Potential pulse step (Es) dependence on peak current for 1–9 mV. Instrumental parameters: ΔE = 30 mV, tw, tp = 10 ms. Stirring rate, 500 rpm.

**Figure 4:**
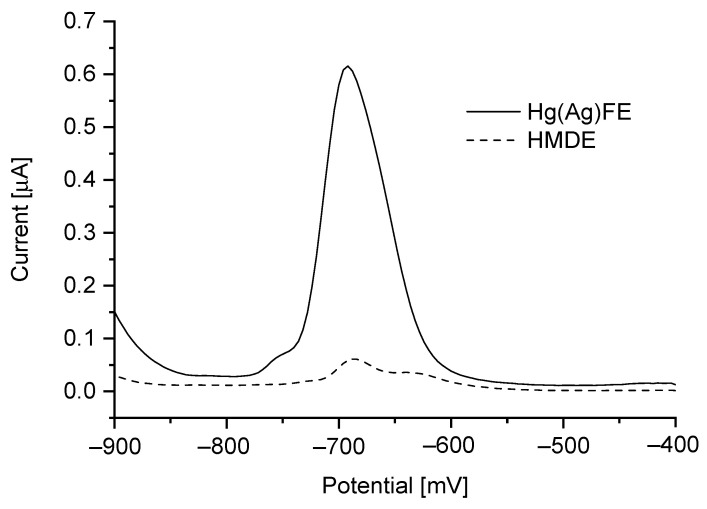
Rifampicin peaks obtained on two types of electrodes: HMDE (1.6 mm^2^) and Hg(Ag)FE (5.7 mm^2^) in 0.1 M CH_3_COOH with the same instrumental parameters applied.

Considering SNR value, the best results were obtained for an amplitude of 30 mV, the selected pulse amplitude was applied for further studies. In the case of potential step amplitude, the rifampicin peak current showed significant growth up to 9 mV with significant growth of the background current (Figure 3). Due to the limitation of measuring data by applying step potential above 4 mV this value was selected for further research. The range of waiting and sampling time were tested from 10 ms to 60 ms, and the best results were obtained with 10 ms for each of them. In order to reach the lowest RIF detection limit, two types of mercury electrodes were chosen for comparison. The voltammograms of RIF obtained on HMDE and Hg(Ag)FE were investigated, compared and showed in Figure 4. The calculated current value of rifampicin peak, taking into account different surface areas of electrodes was 2.8 times higher for Hg(Ag)FE than for HMDE in the same condition.

### 3.3. Influence of the Surface Area of the Hg(Ag)FE Electrode on Rifampicin Peak

**Figure 5:**
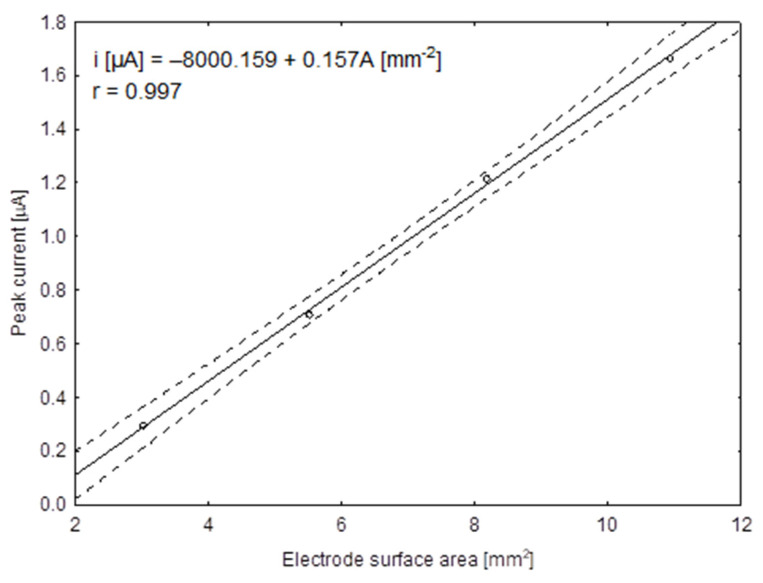
The dependence of peak current on Hg(Ag)FE surface area from 2.7 to 10.7 mm^2^ obtained for 0.2 µgmL^−1^ of RIF in 0.1 M CH_3_COOH solution.

The solid electrode surface areas are usually greater than those of conventional mercury drop electrodes. However, when using the Hg(Ag)FE as a working electrode, its surface can be easily adjusted over a wide range. The rifampicin peak heights were linearly dependent on the surface area of the working electrode (Figure 5). The linear relationship was described by following parameters: slope, 0.175 ± 0.004 [µA mm^−2^]; intercept, −0.240 ± 0.027 [µA] and Pearson correlation coefficient, r = 0.9996. The size of the working electrode of 10.9 mm^2^ was selected for further studies. 

### 3.4. Interferences

**Figure 6:**
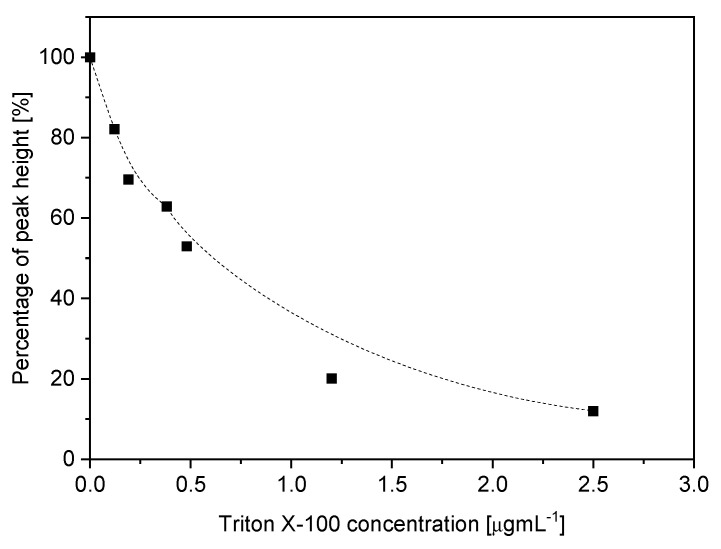
Influence of active-surface compound (Triton X–100) addition in range 0.5–2.5 mgL^–1^ on rifampicin peak height in 0.1 M CH_3_COOH. Instrumental parameters: ΔE = 30 mV, Es = 4 mV, tw, tp = 10 ms. Stirring rate, 500 rpm.

As a source of strong interferences in voltammetric methods, the surface-active compounds are usually suggested. Therefore, some common excipients found in pharmaceutical formulations (silicium oxide, carboxymethyl starch, sodium laureth sulfate, talc and magnesium stearate) and a nonionic surface-active compound (Triton X-100) were investigated. Based on the obtained results it was shown that tested excipients do not significantly affect RIF peaks. Whereas Triton X-100 solution caused reduce the signal by 47%, 80% and 87% for 0.5 mgL^−1^, 1.2 mgL^−1^ and 2.5 mgL^−1^, respectively (Figure 6). Triton concentration above 1 mgL^−1^ causes peak drift to a more negative potential. It was suggested that hundreds of times concentration of surface-active compounds such as Triton X-100 does not significantly affect the quantitative analysis of RIF. The presence of isoniazid, as one of the components of Rifamazid^®^ was studied. Isoniazid in an acidified electrolyte solution (0.1 M CH_3_COOH) does not interfere and was studied before [28]. 

### 3.5. Analytical Performance

**Figure 7:**
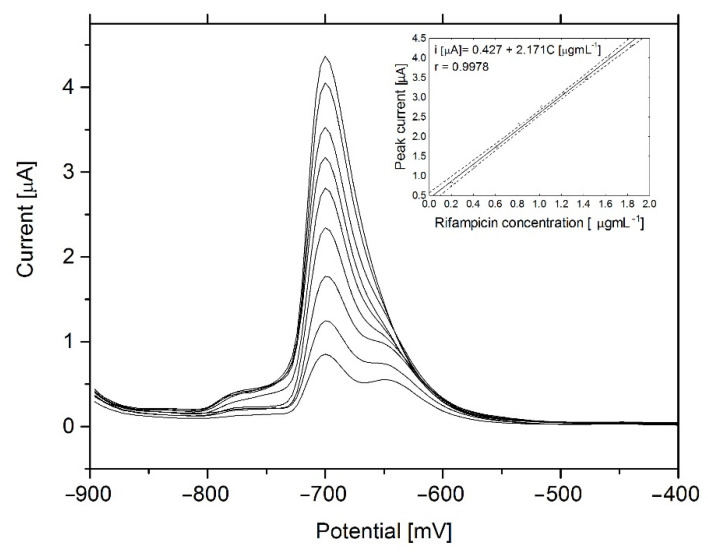
Linearity range of voltammetric determination of rifampicin 0.2–1.8 µgmL^−1^. The electrode area was 9.7 mm^2^. Instrumental parameters: ∆E = 30 mV, E s = 4 mV, tw, tp = 10 ms. Stirring rate, 500 rpm.

The linearity range of up to 250 µgmL^−1^ was tested, but the smaller range was selected for further studies. The DP cathodic voltammograms of RIF for the 0.2–1.8 µgmL^−1^ concentration range without preconcentration step are presented in Figure 7. In this concentration range the slope for regression line is 2.17 ± 0.05 [µAµg^−1^mL] with correlation coefficient r = 0.996. The occurrence of the autocorrelation of residues was assessed using the Durbin-Watson test. In this case, the value of Durbin-Watson’s statistics is d = 1.24, which is between the upper (du = 1.32) and bottom (dl = 0.82) threshold, so the test does not determine of correlation of the random component. Next, according to the method of Lagrange multipliers, the autocorrelation of the random component was not shown. The normal distribution of the residuals was examined by the Shapiro-Wilk test, (W = 0.964 > W*0.05 = 0.829; *p* = 0.84 > 0.05) and it was found that there are no bases for rejecting the hypothesis of a normal distribution of the random component. Therefore, heteroscedasticity of the random component was verified by Bartlett’s test and the results are χ^2^ = 0.009, df = 1, *p* = 0.92 > 0.05 which shown that variance of the residuals in the model is constant.

The estimation of the limits of detection (LOD) and quantification (LOQ) was based on SNR value of 3:1 and 10:1, respectively. Base on the obtained results of the short time analysis (without pre-concentration step), it is possible to achieve LOD = 0.12 µgmL^−1^ and LOQ = 0.4 µgmL^−1^ for RIF. The comparison of sensitivity obtained for RIF using other analytical techniques from available literature was shown in Table 1. Obtained results are positioned within the best results using simple, low-cost instrumentation and short-time analysis. 

The repeatability of the method at a concentration level of the analyte as low as 10 µgmL^−1^, expressed as RSD is 4.16% for six consecutive measurements. Bulk pharmaceutical samples (described in Section 2.4) and samples enriched with 75, 150 and 225 mg of RIF were used for the precision and recovery tests of the method. Quantitative analysis of the RIF samples carried out using the Hg(Ag)FE and the standard addition technique. The recovery of rifampicin in Rifamazid ranged from 98.1–102.9% and Table 2. Results of rifampicin determination in pharmaceuticals formulation are presented in Table 3. 

## 4. Conclusions

The presented DPV method for the electrochemical determination of rifampicin using a cylindrical silver-based mercury film electrode (Hg(Ag)FE), allows determining rifampicin at trace level, without preconcentration step. Determination of rifampicin under optimized condition exhibited high sensitivity (LOD = 0.12 µgmL^−1^, LOQ = 0.40 µgmL^−1^), wide linearity range (0.2–250 μgmL^−1^), good precision (RSD = 4.3%), and good recovery. The repeatability of the method, with each measurement carried out at a fresh surface of the working electrode, is satisfactory and equals RSD = 4.16%. The proposed method is applicable for the rifampicin determination in pharmaceuticals. The obtained results were in good agreement with the content of API declared by the manufacturer, even in the presence of common excipients and isoniazid. The obtained results confirm that the developed method might be a useful tool for the quality control of pharmaceutical products and drug monitoring, whereas Hg(Ag)FE could be incorporated into portable voltammetric sensor systems. 

## Figures and Tables

**Table 1:** The comparison of the sensitivity of selected analytical techniques for rifampicin.

Analytical Technique	LOD [µgmL^−1^]	LOQ [µgmL^−1^]	Reference
UHPLC–UV	1.9	2.9	[13]
MEKC	4.5	14.9	[14]
LC–MS/MS	0.1 *	0.1 *	[16]
Fluorimetry	0.25	0.75	[17]
DPV:HMDE	0.13	0.4	[30]
DPV:Hg(Ag)FE	0.12	0.4	This work

* LLOQ—lower limit of quantification; UHPLC—ultra-high-performance liquid chromatography; MEKC—micellar electrokinetic chromatography; LC-MS/MS—high-performance liquid chromatography-tandem mass spectrometry.

**Table 2:** Recovery of rifampicin determination in Rifamazid^®^.

	Added [mg]	Found [mg]	Recovery [%]
Rifampicin	-	149.5 ± 6.4	-
	75	221.9 ± 8.2	98.8
	150	308.1 ± 11.7	102.9
	225	367.5 ± 12.6	98.1

**Table 3:** Rifampicin determination in pharmaceuticals formulation.

Sample	Nominal [mg]	Found [mg]	µ [mg]	Recovery [%]	RSD [%]	SD
Rifamazid^®^ 150 mg rifampicin 100 mg isoniazid	150	145.2 152.4 141.1 145.8 155.4 157.3	149.5	99.7	4.3	6.4
Rifampicyna TZF 150 mg rifampicin	150	142.6 144.7 146.5 147.2 151.5 158.8	148.5	99.0	3.9	5.8

## Data Availability

The data is available from the authors upon reasonable request.

## References

[1] Zumla A., Raviglione M., Hafner R., von Reyn C.F. (2013). Tuberculosis. N. Engl. J. Med..

[2] Trestioreanu A.Z., Fraser A., Gafter-Gvili A., Paul M., Leibovici L. (2013). Antibiotics for preventing meningococcal infections. Cochrane Database Syst. Rev..

[3] Lee M.Y., Bourgeois S., Almouazen E., Pelletier J., Renaud F., Fessi H., Kodjikian L. (2016). Microencapsulation of rifampicin for the prevention of endophthalmitis: In vitro release studies and antibacterial assessment. Int. J. Pharm..

[4] Ray J., Gardiner I., Marriott D. (2003). Managing antituberculosis drug therapy by therapeutic drug monitoring of rifampicin and isoniazid. Intern. Med. J..

[5] Alsultan A., Peloquin C.A. (2014). Therapeutic Drug Monitoring in the Treatment of Tuberculosis: An Update. Drugs.

[6] Moussa L.A., El Bouazzi O., Serragui S., Tanani D.S., Soulaymani A. (2016). Rifampicin and isoniazid plasma concentrations in relation to adverse reactions in tuberculosis patients: A retrospective analysis. Ther. Adv. Drug Saf..

[7] WHO (2020). Global Tuberculosis Report 2020.

[8] Visca D., Ong C., Tiberi S., Centis R., D’Ambrosio L., Chen B., Mueller J., Duarte R., Dalcolmo M., Sotgiu G. (2021). Tuberculosis and COVID-19 interaction: A review of biological, clinical and public health effects. Pulmonology.

[9] Migliori G.B., Thong P.M., Akkerman O., Alffenaar J.-W., Álvarez-Navascués F., Assao-Neino M.M., Bernard P.V., Biala J.S., Blanc F.-X., Bogorodskaya E.M. (2020). Worldwide Effects of Coronavirus Disease Pandemic on Tuberculosis Services, January–April 2020. Emerg. Infect. Dis..

[10] Shewiyo D., Kaale E., Risha P., Dejaegher B., Smeyers-Verbeke J., Heyden Y.V. (2012). Optimization of a reversed-phase-high-performance thin-layer chromatography method for the separation of isoniazid, ethambutol, rifampicin and pyrazinamide in fixed-dose combination antituberculosis tablets. J. Chromatogr. A.

[11] Rageh A.M.I.M., Mohamed F.A., Atia N.N., Botros S.M. (2015). Simultaneous Densitometric Determination of First Line Anti-TB Drugs in Binary, Ternary, and Quaternary Mixtures. J. Liq. Chromatogr. Relat. Technol..

[12] Strock J., Nguyen M., Sherma J. (2015). Transfer of Minilab TLC Screening Methods to Quantitative HPTLC-Densitometry for Pyrazinamide, Ethambutol, Isoniazid, and Rifampicin in a Combination Tablet. J. Liq. Chromatogr. Relat. Technol..

[13] Franco P.H.C., Chellini P.R., Oliveira M.A.L., Pianetti G.A. (2017). Simultaneous Determination of First-Line 4-FDC Antituberculosis Drugs by UHPLC–UV and HPLC–UV: A Comparative Study. J. AOAC Int..

[14] Iriminescu D., Cârcu-Dobrin M., Hancu G., Mircia E., Kelemen H., Rusu A., Tilinca M. (2016). Simultaneous determination of isoniazid an drifampicin by micellar electrokinetic chromatography. Stud. Univ. Vasile Goldis Arad. Ser. Stiint. Vietii.

[15] Marcellos L.F., Faria A.F., Souza M.V.N., Almeida M.R., Sabin G.P., Poppi R., Oliveira M.A.L. (2012). Simultaneous analysis of first-line anti-tuberculosis drugs in tablets by UV spectrophotometry compared to capillary zone electrophoresis. Open Chem..

[16] Grégoire M., Leroy A., Bouquié R., Malandain D., Dailly E., Boutoille D., Renaud C., Jolliet P., Caillon J., Deslandes G. (2016). Simultaneous determination of ceftaroline, daptomycin, linezolid and rifampicin concentrations in human plasma by on-line solid phase extraction coupled to high-performance liquid chromatography–tandem mass spectrometry. J. Pharm. Biomed. Anal..

[17] Liu Z., Yin P., Gong H., Li P., Wang X., He Y. (2012). Determination of rifampicin based on fluorescence quenching of GSH capped CdTe/ZnS QDs. J. Lumin..

[18] Kawde A.-N., Temerk Y., Farhan N. (2014). Adsorptive stripping voltammetry of antibiotics rifamycin SV and rifampicin at renewable pencil electrodes. Acta Chim. Slov..

[19] Asadpour-Zeynali K., Mollarasouli F. (2017). Novel electrochemical biosensor based on PVP capped CoFe_2_O_4_ @CdSe core-shell nanoparticles modified electrode for ultra-trace level determination of rifampicin by square wave adsorptive stripping voltammetry. Biosens. Bioelectron..

[20] Chokkareddy R., Bhajanthri N.K., Redhi G.G. (2017). A Novel Electrode Architecture for Monitoring Rifampicin in Various Pharmaceuticals. Int. J. Electrochem. Sci..

[21] Beni V., Ogurtsov V.I., Bakunin N.V., Arrigan D.W., Hill M. (2005). Development of a portable electroanalytical system for the stripping voltammetry of metals: Determination of copper in acetic acid soil extracts. Anal. Chim. Acta.

[22] Baś B., Kowalski Z. (2002). Preparation of Silver Surface for Mercury Film Electrode of Prolonged Analytical Application. Electroanalysis.

[23] Pecková K., Vrzalová L., Bencko V., Barek J. (2009). Voltammetric and amperometric determination of N-nitroso antineoplastic drugs at mercury and amalgam electrodes. Collect. Czechoslov. Chem. Commun..

[24] Baś B., Jakubowska M., Górski Ł. (2011). Application of renewable silver amalgam annular band electrode to voltammetric determination of vitamins C, B1 and B2. Talanta.

[25] Brycht M., Skrzypek S., Guzsvány V., Berenji J. (2013). Conditioning of renewable silver amalgam film electrode for the characterization of clothianidin and its determination in selected samples by adsorptive square-wave voltammetry. Talanta.

[26] Piech R., Paczosa-Bator B. (2013). Sensitive and fast determination of papaverine by adsorptive stripping voltammetry on renewable mercury film electrode. Open Chem..

[27] Smajdor J., Piech R., Paczosa-Bator B. (2017). Voltammetric Determination of Drospirenone on Mercury Film Electrode. J. Electrochem. Soc..

[28] Szlósarczyk M., Piech R., Paczosa-Bator B., Maslanka A., Opoka W., Krzek J. (2012). Voltammetric Determination of Isoniazid using Cyclic Renewable Mercury Film Silver Based Electrode. Pharm. Anal. Acta.

[29] Górska A., Paczosa-Bator B., Piech R. (2020). Highly Sensitive Levodopa Determination by Means of Adsorptive Stripping Voltammetry on Ruthenium Dioxide-Carbon Black-Nafion Modified Glassy Carbon Electrode. Sensors.

[30] Alonso-Lomillo M.A., Domínguez-Renedo O., Arcos-Martínez J. (2002). Optimization Procedure, Applying the Experimental-Design Methodology, for the Determination of Rifampicin after Metal Complexation by Differential Pulse Adsorptive Stripping Voltammetry. Helv. Chim. Acta.

